# Nutrient Limitation on Ecosystem Productivity and Processes of Mature and Old-Growth Subtropical Forests in China

**DOI:** 10.1371/journal.pone.0052071

**Published:** 2012-12-20

**Authors:** Enqing Hou, Chengrong Chen, Megan E. McGroddy, Dazhi Wen

**Affiliations:** 1 Key Laboratory of Vegetation Restoration and Management of Degraded Ecosystems, South China Botanical Garden, Chinese Academy of Sciences, Guangzhou, China; 2 Environmental Futures Centre, Griffith School of Environment, Griffith University, Nathan, Queensland, Australia; 3 Department of Environmental Sciences, NASA/University of Virginia, Charlottesville, Virginia, United States of America; 4 University of Chinese Academy of Sciences, Beijing, China; Federal University of Rio de Janeiro, Brazil

## Abstract

Nitrogen (N) is considered the dominant limiting nutrient in temperate regions, while phosphorus (P) limitation frequently occurs in tropical regions, but in subtropical regions nutrient limitation is poorly understood. In this study, we investigated N and P contents and N:P ratios of foliage, forest floors, fine roots and mineral soils, and their relationships with community biomass, litterfall C, N and P productions, forest floor turnover rate, and microbial processes in eight mature and old-growth subtropical forests (stand age >80 yr) at Dinghushan Biosphere Reserve, China. Average N:P ratios (mass based) in foliage, litter (L) layer and mixture of fermentation and humus (F/H) layer, and fine roots were 28.3, 42.3, 32.0 and 32.7, respectively. These values are higher than the critical N:P ratios for P limitation proposed (16–20 for foliage, ca. 25 for forest floors). The markedly high N:P ratios were mainly attributed to the high N concentrations of these plant materials. Community biomass, litterfall C, N and P productions, forest floor turnover rate and microbial properties were more strongly related to measures of P than N and frequently negatively related to the N:P ratios, suggesting a significant role of P availability in determining ecosystem production and productivity and nutrient cycling at all the study sites except for one prescribed disturbed site where N availability may also be important. We propose that N enrichment is probably a significant driver of the potential P limitation in the study area. Low P parent material may also contribute to the potential P limitation. In general, our results provided strong evidence supporting a significant role for P availability, rather than N availability, in determining ecosystem primary productivity and ecosystem processes in subtropical forests of China.

## Introduction

Nitrogen (N) and phosphorus (P) have both been shown to control the rates of ecosystem processes and primary productivity in both aquatic and terrestrial ecosystems [1]–[3]. Global pattern analysis of carbon (C):N:P stoichiometry in foliage and litter supports the hypothesis that N is the major limiting nutrient in temperate regions, while P tends to limit ecosystem productivity and processes in the tropical regions [4]–[7]. These analyses are generally consistent with the nutrient addition experiments or C:N:P stoichiometry studies at a local or regional scales [8]–[12], and well explained by variation in climate conditions (e.g. temperature) and soil types [7], [12]–[14]. According to this global pattern, subtropical forests are likely to be co-limited by N and P. However, this supposition has rarely been tested.

Since the beginning of the industrial revolution, human activities (N fertilizer application and burning of fossil fuels) have doubled the N input into the terrestrial ecosystems [15], [16]. Although anthropogenic P inputs (mainly as fertilizers) to the biosphere also increased fourfold in the period from 1950s to 1980s and remained more or less constant since 1989, the primary P inputs are mostly confined in agricultural soils and tend to remain and accumulate in crop soils [17]. The greater mobility and biological availability of N in the atmosphere are causing the imbalance supply between N and other mineral nutrients (especially P) in natural ecosystems [17], [18], which is likely to transform N-limited ecosystems to P-limited ecosystems [16], [19]. In a comprehensive study of nutrients on phytoplankton nutrient limitation in high- and low-N deposition lakes in Norway, Sweden, and Colorado, United States, Elser et al. (2009) found that continued anthropogenic N input increased the stoichiometric ratio of N and P in these lakes, resulting in a shift from N-limitation to P-limitation in high-N deposition lakes [16]. The imbalance of nutrient supply is likely to affect ecosystem productivity and processes and the carbon sequestration potential of terrestrial ecosystems [17], [19], [20].

China has 0.97 million km^2^ of subtropical and tropical forests, which represent 62% of the country’s total forested area, and play an important role in maintaining biodiversity and ecological equilibrium, sequestering atmospheric C, and providing important ecological services for social development [21], [22]. However, these tropical and subtropical forests in the southern part of China, are generally close to or surrounded by large industrial and/or economic zones. Annual N deposition rate ranging from 18 to 53 kg N ha^−1^ yr^−1^ were reported at several long-term monitoring stations in tropical and subtropical forests [22], comparable to the highest levels of N deposition occurring in Europe [23], [24]. Recent studies found that the understory plants generally showed no or even negative responses to experimental N additions (50, 100 and 150 kg N ha^−1^ yr^−1^) in three mature and old-growth forests at the Dinghushan Biosphere Reserve, south China [25], [26]. Nutrients other than N, were proposed as the primary constraint on plant growth at these forest sites with P being the mostly likely candidate [26]. However, direct evidence is still lacking, though one recent study reported a significant increase in litterfall production after experimental P addition (150 kg P ha^−1^ yr^−1^) at these three forests [27].

While fertilization studies are the gold standard for determining the nature of nutrient limitation [28], [29], they are difficult to do well in forest ecosystems [2], and, in many cases, after several years of study the results are still unclear [31]–[33]. It may be a question of how much fertilizer to add, as Chapin (1986) suggested [30], or if the nutrient limitation is ultimate, it may take decades or more for species replacement to happen in forest ecosystems and thus delaying measurable results [18], [31]. The critical N:P ratio for biomass was shown to work well indicating the limiting nutrient in European wetland ecosystems [34], but is poorly supported in some other terrestrial ecosystems [35], [36].

In this study, we investigated the N and P status of foliage, forest floors, fine roots and mineral soil, as well as microbial properties of the forest floors of eight forests in subtropical China. We used regression analysis, to study the relationships between rates of ecosystem productivity and nutrient cycling and N and P availability in these forests. We hypothesized that these selected parameters were more strongly related to P availability than N availability, due to the historically high rates of atmospheric N deposition [22].

## Methods

### Site Description

**Figure 1:**
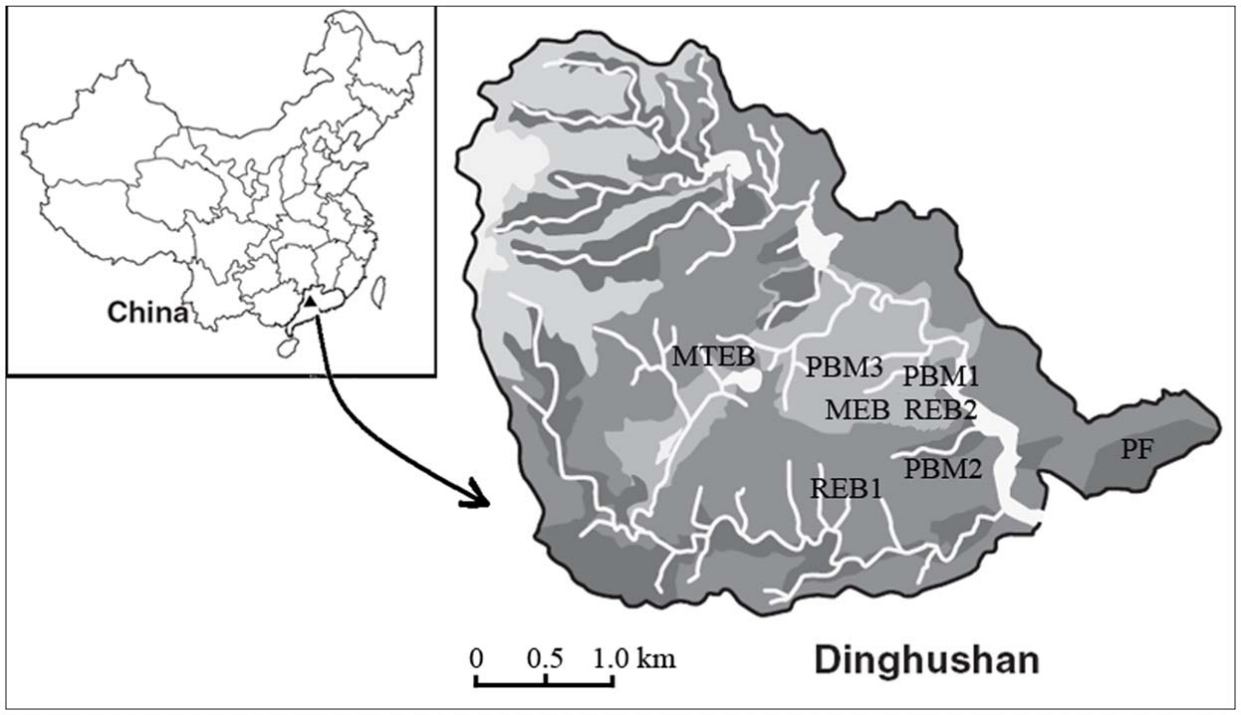
Location of eight study forest sites.

The research was conducted in the Dinghushan Biosphere Reserve, located in the middle of Guangdong province in southern China (112°31′ E to 112°34′ E, 23°09′ N to 23°12′ N; Figure 1). The Reserve covers an area of 1155 ha, and has a typical subtropical monsoon climate. The entire Reserve has 1843 plant species identified and documented [37]. Mean annual temperature at the site is 21°C, and mean annual precipitation is 1900 mm [38]. Nearly 80% of the precipitation falls in the wet season (from April to September) and 20% in the dry season (from October to March) [38]. Elevation ranges from 10 to 1000 m above sea level. The forest soil has developed from Devonian sandstone and shale during the Holocene (<15 kyr) [39]. Soils are Ferralsols according to the FAO classification, with a pH value ranging from 3.8 to 4.9 [40], [41]. The annual rate of atmospheric N deposition was approximately 46 kg N ha^−1^ yr^−1^ between 1989 and 2007 [42].

The basic site information for the eight selected forest communities is summarized in Table S1. These eight communities cover all major forest types in DHSBR, representing five typical forest types in subtropical China [43]. They differ in tree species composition, stand age and topography (Table S1). Four of them (PF: pine forest; PBM1, 2 and 3, pine and broadleaved mixed forest 1, 2 and 3) are mature forests (about 80 years old), while the other four communities (REB1 and 2, ravine evergreen broadleaved forest 1 and 2; MEB, monsoon evergreen broadleaved forest; and MTEB, mountainous evergreen broadleaved forest) are old-growth forests (>100 year old) and were regional or topographical climax forests (Table S1) [44]. Seven of the forests have been protected from human disturbance since their establishment. The PF is the exception, it had been disturbed mainly by the harvest of understory vegetation and litter for fuel by local residents between 1950s and 1990s [45], whilst the community has remained and dominated by *Pinus massoniana*, of which biomass was about 90% of the total community biomass [46].

### Sampling and Sample Preparation

During 15th – 18th October, 2010, we sampled foliage, forest floors, fine roots and soil at each of the eight study sites. At each site, mature and healthy foliage was sampled from three major tree species which were listed in Table S1. For each species, we collected foliar samples from four individuals.

At each location, four subplots (20×20 m^2^) were randomly set up with a distance of at least 10 m between them. In each subplot, 3 small sampling areas (20×20 cm^2^) were randomly located with the constraints that they were 1–2 m away from the nearest tree (diameter at breast height ≥5 cm) and at least 5 m from its nearest neighbor. All fine forest floor materials within the sampling area were collected, including leaf litter, and senesced branches, bark, flowers and fruits with diameters ≤1 cm. Forest floor materials were carefully separated into two layers (L layer, litter layer; F/H layer, mixture of fermentation layer and humus layer) in the field. After forest floor materials were sampled, a soil profile was excavated at the same area. Mineral soil from the 0–15 cm depth was sampled by 3 successive cutting rings (Height 5 cm, Volume 100 cm^3^) from top to bottom (each 5 cm depth by one cutting ring). By using a cutting ring to sample soil, we also measured the bulk density at the same time of soil sampling. The three forest floor samples from the same layer in each subplot were bulked together as one composite sample (one composite L layer sample and one composite F/H layer sampler per subplot). Nine soils (3 cutting rings per area×3 areas) of each subplot were bulked together as one mineral soil sample. Both forest floor and soil samples were stored at 4°C in the refrigerator within 4 h after sampling.

Leaves were directly oven-dried at 65°C for 72 h prior to grinding for determination of total N and P concentrations. For forest floor materials, the fresh weight (w1, unit: g) was recorded and then the sample was mixed well. A subsample was oven-dried at 65°C for 72 h for the determination of dry weight transfer coefficient (t, proportion of dry weight over the fresh weight). The forest floor biomass was calculated by the equation followed:

L layer (or F/H layer) forest floor biomass (g/cm^2^) = (w1×t)/(400 cm^2^×3).

The unit was converted later to Mg/ha and shown in the results. After t was determined, the oven-dried sample was ground for the determination of total N and P concentrations. Another subsample was taken and cut into 2–4 mm pieces and stored at 4°C prior to the determination of microbial biomass C concentration, respiration and β-glucosidase activity.

For soil samples, fresh weight (W1, unit: g) was recorded and the sample was mixed well. Stones with diameter >4 mm were picked out during the sieving (4 mm mesh) and weighed (W2, unit: g). A subsample of the sieved soil was air-dried for 2 weeks prior to grinding and determination of soil nutrient concentration. A subsample was used for the soil dry weight transfer coefficient (T; proportion of dry weight after over-dried in fresh weight) determination by oven-dried at 105°C for 72h. The bulk density was calculated by equations followed:

Bulk density (g/cm^3^) = W2+ (W1–W2)×T/(100 cm^3^×9).

The remaining soil was weighed and stored at 4°C for the determination of microbial properties (data not shown here) and fine roots (diameter ≤2 mm) collection. Fine roots retained on a 0.6 mm screen were collected and dried at 65°C for 72 h and then weighed for the fine root biomass calculation (data not shown here). Fine roots were finely ground for measurements of total N and P concentrations.

### Analytical Methods

Microbial biomass C concentration in the L and F/H layers was determined by a fumigation-extraction (1∶25) method using an *E_C_* factor of 2.64 [47]. Microbial respiration was measured by using the incubation method. In brief, 2 g of fresh forest floor materials with moisture adjusted to 60% of the field capacity was incubated aerobically in a 1–L sealed plastic jar at room temperature (ranging from approximate 15°C at night time to 25°C at day time). All CO_2_ evolved was trapped in 0.1 M NaOH and measured by acid titration (0.1 M HCl) after 1, 3, 7, 14, 21 and 28 days. The activity of β-glucosidase (EC 3.2.1.21) was analyzed following the procedure of Alef and Nannipieri (1995) [48], except that a fresh weight of 0.5 g was used for our forest floor samples.

Total N concentrations of foliage, L and F/H layers, fine roots and soil were all determined using an Isoprime isotope ratio mass spectrometer with a Eurovector elemental analyzer (Isoprime-Euro EA 3000). Total P concentrations were all measured using a nitric acid/perchloric acid digestion, followed by the molybdate blue method [49] using a UV–Vis spectrometer (UV1800, Shimadzu, Japan). Soil extractable N concentration was measured as the hot water extractable total N concentration according to method described by Sparling (1998) [50], which was found to be a simple and useful predictor of mineralizable N and plant available N [51], [52]. In brief, 4.0 g air-dried soil was incubated with 20 ml water in a capped test tube at 70°C for 18 h. The test tube was then shaken on an end-to-end shaker for 5 min, and filtered through Whatman 42 filter paper. Total N concentration of the extract was analyzed using a SHIMADZU TOC-_VCPH/CPN_ analyser (Kyoto, Japan). Soil extractable P concentration was calculated as the sum of inorganic P concentration sequentially extracted by 1.0 M NH_4_Cl, 0.1 M NH_4_F and 0.1 M NaOH following the P fractionation scheme of McDowell and Condron (2000) [53]. Concentrations of inorganic P in the extracts of 0.1 M NH_4_F and 0.1 M NaOH were determined by the molybdate blue method [49] using the same UV–Vis spectrometer mentioned above; while concentrations of inorganic P in the extracts of 1.0 M NH_4_Cl were too low for the molybdate blue method and thus determined by the malachite green method [54], which works well for the determination of low concentrations of P in soil extracts [54], using the same UV–Vis spectrometer, too.

### Community Biomass and Litterfall C, N and P Productions

Community biomass reported by Liu et al. (2007) [55] and litterfall production reported by Zhou et al. (2007) [44] and Yan et al. (2009) [56] from these forest communities were used to investigate the relationships of community biomass and litterfall C, N and P productions with soil nutrient pools in this study. Litterfall C, N and P productions were calculated by multiplying litterfall production by the C, N and P concentrations in the L layer. Community biomass includes dry weight of whole plant of all trees and shrubs with diameter at breast height ≥1 cm (Table S1). Litterfall production was available for seven of the eight study forests, while not for the PBM3 (Table S1). Methods of the data collection or calculation are described in details in Table S1.

### Forest Floor Turnover Rate

Jenny et al. (1949) [57] and subsequently Olson (1963) [58] proposed that the rate of change in the forest floor biomass (or biomass C) could be used to determine nutrient transfers from the forest floor to the mineral soil in (near-) equilibrium forests. Forests selected in this study are all in or near an equilibrium status except for the PF site, as suggested by the study of long-term change of litterfall production [44]. Here, we calculated the forest floor turnover rate according to Olson (1963) [58]. The calculation formula is:

Forest floor turnover rate (yr^−1^) = litterfall C production/forest floor biomass C.

Forest floor biomass C was the sum of L layer and F/H layer biomass C.

### Statistical Analyses

Since the data are mostly (near-) normal distributed, Pearson correlation analysis and Pearson linear regression technique were used throughout the manuscript. All N:P ratios shown in this study were calculated on a mass basis. All analyses were performed using SPSS version 16.0. Pearson correlation was used to investigate the correlations between N and P concentration and N:P ratio for all plant and soil samples, and was also used to investigate the correlations between nutrient measures of the plant samples and those of the soil samples. Pearson linear regression technique was used to examine the relationships between community biomass, litterfall C, N and P productions, forest floor turnover rate and forest floor microbial properties with nutrient measures of the soil or the forest floors.

According to the foliar N concentration, foliage was divided into two groups (see Figure S1). One was the high N group with foliar N concentration higher than 25 mg/g (sample number = 20), including species of *Gironniera subaequalis* (from the REB2), *Ormosia fordiana* (REB1), *Caryota ochlandra* (REB2), *Euodia lepta* (PF) and *Sterculia lanceolata* (REB2); the other was low N group with foliar N concentration lower than 25 mg/g (sample number = 76), including the other eight species (Table S4 and Figure S1). For plants of the same species, only four species with a sample number ≥8 were selected for the correlation analysis. To be consistent with respect to units, when community biomass and litterfall C, N and P productions were regressed against the soil nutrients, pools were used; while for all other correlation and regression analysis, concentrations were used.

## Results

### Community Biomass and Litterfall Productions in Relation to the Nutrient Measures

**Figure 2:**
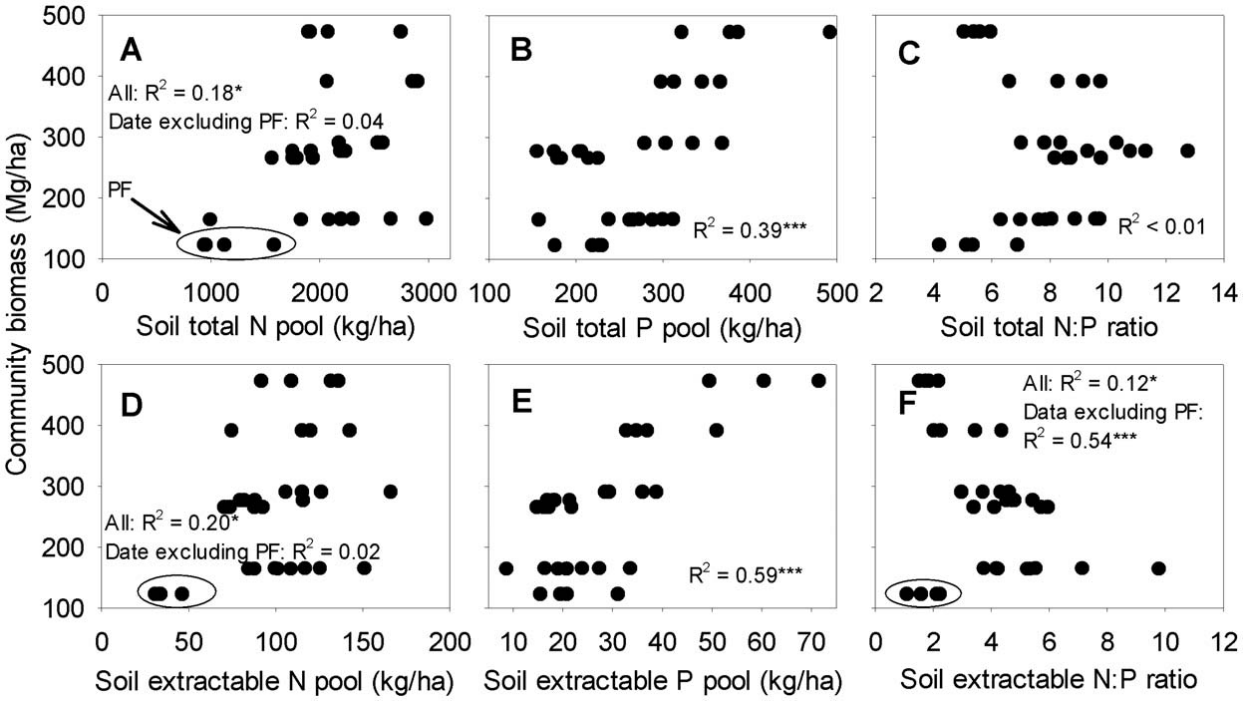
Relationships between community biomass and soil nutrient measures. PF indicates the pine forest. * *P*<0.05; *** *P*<0.001.

Community biomass was positively related to both soil total P (R^2^ = 0.39, *P*<0.001; Figure 2B) and extractable P pools (R^2^ = 0.59, *P*<0.001; Figure 2E). The relationships with soil total N (R^2^ = 0.18, *P*<0.05; Figure 2A) and extractable N (R^2^ = 0.20, *P*<0.05; Figure 2D) pools were also significant but mainly caused by the inclusion of the prescribed PF site. When the PF site was excluded, community biomass was not related to either the soil total N (R^2^ = 0.04, *P*>0.05; Figure 2A) or the extractable N pool (R^2^ = 0.02, *P*>0.05; Figure 2D). Community biomass tended to increase with increasing soil total N:P ratio when community biomass was low (<300 Mg/ha), while increasing with decreasing soil total N:P ratio when community biomass was high (≥300 Mg/ha; Figure 2C). Overall, community biomass was not related to the soil total N:P ratio (R^2^<0.01, *P*>0.05; Figure 2C). Community biomass was negatively related to soil extractable N:P ratio when the PF site was excluded from the analysis (R^2^ = 0.54, *P*<0.001; Figure 2F).

**Figure 3:**
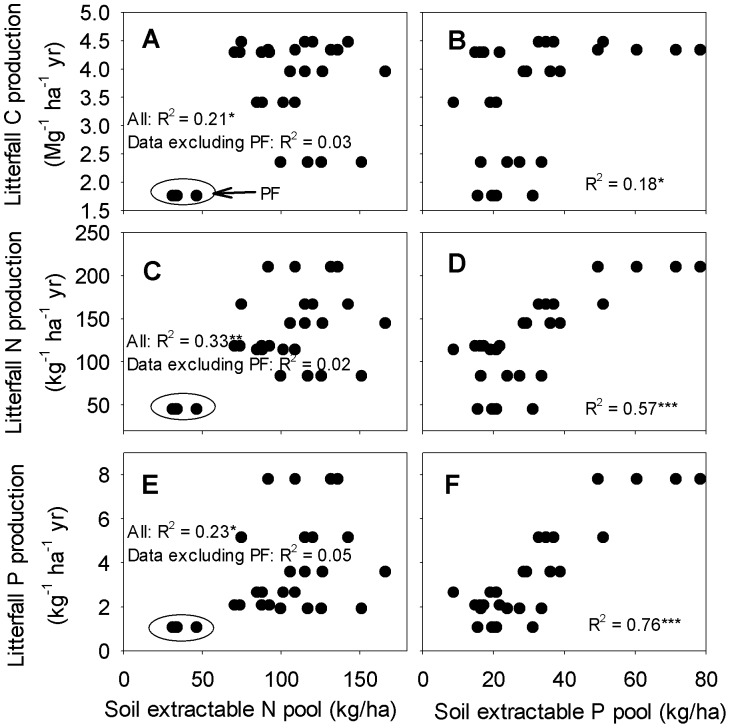
Relationships between litterfall C, N and P productions and soil nutrient measures. PF indicates the pine forest. ** *P*<0.01; *** *P*<0.001.

Litterfall C, N and P productions were all positively related to the soil extractable P pool (R^2^ = 0.18, 0.57 and 0.76, respectively, *P*<0.05; Figures 3B, D and F). Similar to the community biomass, the relationships between litterfall C, N and P productions and the soil extractable N pool were significant for all sites (R^2^ was 0.21, 0.33 and 0.23, respectively, *P*<0.05; Figures 3A, C and E), but none was significant if the PF site was excluded (R^2^ = 0.03, 0.02 and 0.05, respectively, *P*>0.05; Figures 3A, C and E). The patterns for litterfall C, N and P productions with soil total fractions and soil extractable fractions were similar (data not shown). Moreover, litterfall C, N and P productions were not related to the soil total N:P ratio (data not shown).

### Forest Floor Turnover Rates in Relation to the Nutrient Measures

**Figure 4:**
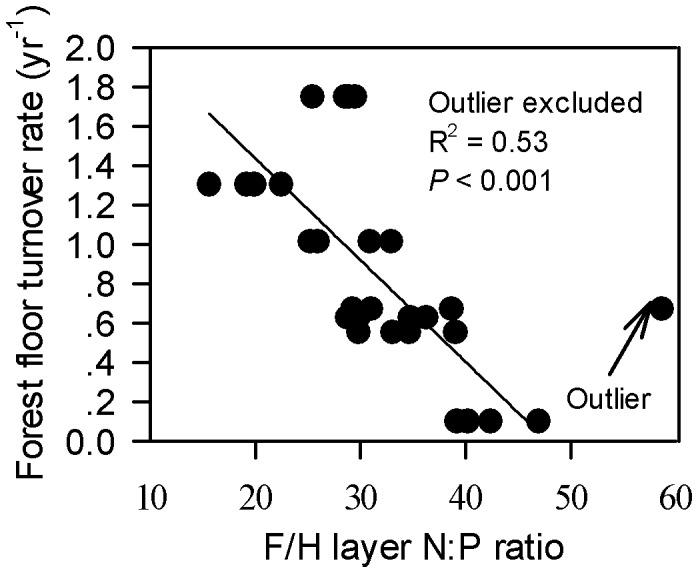
Relationships between forest floor turnover rate with F/H layer N:P ratio.

Forest floor turnover rate (yr^−1^) ranged from 0.10 to 1.75 among the seven study communities for which it was calculated (no data for PBM3), with an average value of 0.86 (Table S2). As summarized in the Table 1, forest floor turnover rate was positively related to the P concentration in both L and F/H layers (L layer: R^2^ = 0.35, *P*<0.001; F/H layer: R^2^ = 0.40, *P*<0.001), while only weakly related to the N concentration in the L layer (R^2^ = 0.18, *P*<0.05). It was also negatively related to the N:P ratio in both L and F/H layers (L layer: R^2^ = 0.19, *P*<0.05; F/H layer: R^2^ = 0.40, *P*<0.001). When one outlier was excluded, the relationship of forest floor turnover rate with the F/H layer N:P ratio was even stronger (R^2^ = 0.53, *P*<0.001; Figure 4).

**Table 1:** Summary of regressions of forest floor turnover rates against nutrient measures of forest floors.

Parameter	Nutrient measure	Regression	R^2^	Significance
**Forest floor turnover rate (yr** ^−**1**^ **)**				
L layer	N conc. (mg/g)	Rate = 0.067(N conc.) –0.226	0.18	<0.05
	P conc. (mg/g)	Rate = 1.729(P conc.) +0.012	0.35	<0.001
	N:P ratio	Rate = −0.019(N:P ratio) +1.638	0.19	<0.05
F/H layer	N conc. (mg/g)			NS
	P conc. (mg/g)	Rate = 2.70(P conc.) –0.36	0.4	<0.001
	N:P ratio	Rate = −0.036(N:P ratio) +2.025	0.4	<0.001
**Microbial biomass C conc. (mg/g)**				
L layer	N conc. (mg/g)			NS
	P conc. (mg/g)			NS
	N:P ratio			NS
F/H layer	N conc. (mg/g)	Microbial C = 0.94(N conc.) − 1.46	0.16	<0.05
	P conc. (mg/g)	Microbial C = 30.7(P conc.) − 2.25	0.34	<0.001
	N:P ratio			NS
**Microbial respiration (µg CO_2_-C g** ^−**1**^ ** h** ^−**1**^ **)**				
L layer	N conc. (mg/g)			NS
	P conc. (mg/g)	Respiration = 24.6(P conc.) +21.1	0.48	<0.001
	N:P ratio	Respiration = −0.32(N:P ratio) +45.0	0.37	<0.001
F/H layer	N conc. (mg/g)			NS
	P conc. (mg/g)			NS
	N:P ratio			NS
**β-glucosidase activity (µg p-nitrophenol g** ^−**1**^ ** h** ^−**1**^ **)**				
L layer	N conc. (mg/g)	β-glucosidase = 19.3(N conc.) − 78.9	0.14	<0.05
	P conc. (mg/g)	β-glucosidase = 626.5(P conc.) − 29.5	0.44	<0.001
	N:P ratio	β-glucosidase = −6.8(N:P ratio) − 518.4	0.23	<0.01
F/H layer	N conc. (mg/g)	β-glucosidase = 8.1(N conc.) +45.0	0.17	<0.05
	P conc. (mg/g)	β-glucosidase = 176.5(P conc.) +75.8	0.16	<0.05
	N:P ratio			NS

n = 32. NS indicates statistically not significant at the level of *P*<0.05.

Microbial biomass C concentration was not related to any nutrient measures in the L layer, but related to the N and P concentrations in the F/H layer, with a stronger relationship with the P concentration (R^2^ = 0.34, *P*<0.001; Table 1) than with the N concentration (R^2^ = 0.16, *P*<0.05; Table 1). Microbial respiration was positively related to the P concentration (R^2^ = 0.48, *P*<0.001; Table 1) and also negatively related to the N:P ratio in the L layer (R^2^ = 0.37, *P*<0.001; Table 1), while not related to any nutrient measures in the F/H layer (Table 1). In the L layer, β-glucosidase activity was more strongly related to the P concentration (R^2^ = 0.44, *P*<0.001; Table 1) than the N concentration (R^2^ = 0.14, *P*<0.05; Table 1), and negatively related to the N:P ratio (R^2^ = 0.23, *P*<0.01; Table 1). In the F/H layer, β-glucosidase activity was poorly related to both N and P concentration (R^2^ = 0.17 and 0.16, respectively, *P*<0.05; Table 1) while not related to the N:P ratio.

### Correlations between Nutrient Measures

For both low N and high N groups of foliage, N and P concentrations were poorly correlated with each other (Table 2 and Figure S1A), and the P concentration was more strongly correlated with N:P ratio than the N concentration (Table 2 and Figures S1B and C). Within a species, N:P ratio was negatively correlated with the P concentration in all species (*P*≤0.033; Table 2), but was not correlated with the N concentration in any species (*P*≥0.072; Table 2). The relationship between foliar N and P concentration of plants of the same species was only significant for one species (*Castanea henryi*; Table 2). For the forest floors, fine roots and soil extractable fraction, N and P concentrations were all correlated with each other (r = 0.385–0.782, *P*≤0.030; Table 2), and N:P ratios were all negatively correlated with the P concentrations (r = −0.610– −0.798, *P*<0.001), while only poorly correlated with the N concentration in the F/H layer (r = 0.380, *P* = 0.032; Table 2). Soil total N:P ratios were positively correlated with soil total N concentration (r = 0.450, *P* = 0.010; Table 2) but not correlated with soil total P concentration (r = −0.201, *P* = 0.270; Table 2). However, the plot of soil total N:P ratio against soil total P concentration showed that the points representing the PF site were distinct from other sites (Figure S2B). Excluding the PF site from analysis, the correlations between the soil total fractions were similar to those of forest floors, fine roots and soil extractable fraction (Table 3 and Figure S2).

**Table 2:** Correlations between nutrient measures in the plant and soil samples.

		N conc. vs. P conc.		N:P ratio vs. N conc.		N:P ratio vs. P conc.	
Sample	n	r	*P*	r	*P*	r	*P*
**Foliage**							
Low N group (<25 mg/g)	76	0.336	0.003	0.435	<0.001	−0.682	<0.001
High N group (≥25 mg/g)	20	−0.449	0.047	0.709	<0.001	−0.929	<0.001
*C. henryi*	16	0.709	0.002	0.085	0.753	−0.634	0.008
*C. concinna*	8	0.250	0.550	0.455	0.257	−0.747	0.033
*P. massoniana*	16	0.458	0.075	0.397	0.128	−0.627	0.009
*S. superba*	20	0.302	0.196	0.411	0.072	−0.740	<0.001
**Forest floor**							
L layer	32	0.630	<0.001	−0.111	0.545	−0.798	<0.001
F/H layer	32	0.385	0.030	0.380	0.032	−0.661	<0.001
**Fine roots**	32	0.782	<0.001	−0.094	0.610	−0.660	<0.001
**0–15 cm mineral soil**							
Total fraction	32	0.747	<0.001	0.450	0.010	−0.201	0.270
Total fraction excluding the PF site	28	0.617	<0.001	0.156	0.428	−0.647	<0.001
Extractable fraction	32	0.530	0.002	0.194	0.288	−0.610	<0.001

For soil total fraction, inclusion and exclusion of the pine forest (PF) site showed distinct results and thus, correlations for all data and data excluding PF were both shown.

As summarized in the Table 3, nutrient measures of all plant materials were generally more strongly correlated with the soil total P and extractable P concentrations than with soil total N and extractable N concentrations. Nitrogen: P ratios of forest floors and fine roots were all negatively correlated with soil total P and extractable P concentrations, while either not or negatively correlated with soil total N or extractable N concentration.

**Table 3:** Correlations of nutrient measures between the plant and soil samples.

	0–15 cm mineral soil					
Plant sample	Total N conc.	Total P conc.	Total N:P ratio	Extractable N conc.	Extractable P conc.	Extractable N:P ratio
**Foliage**						
N conc.	0.235	0.419	−0.313	0.126	0.548	−0.669
P conc.	0.277	0.357	−0.172	0.137	0.442	−0.538
N:P ratio	−0.253	−0.024	−0.399	−0.159	−0.105	−0.073
**L layer forest floor**						
N conc.	0.502**	0.668***	−0.074	0.607***	0.708***	−0.147
P conc.	0.218	0.661***	−0.426*	0.377*	0.833***	−0.562**
N:P ratio	0.040	−0.387*	0.482**	−0.076	−0.523**	0.578**
**F/H layer forest floor**						
N conc.	0.101	0.256	−0.377*	−0.018	0.042	−0.127
P conc.	0.349	0.782***	−0.468**	0.387*	0.787***	−0.604***
N:P ratio	−0.303	−0.550**	0.118	−0.381*	−0.646***	0.477**
**Fine roots**						
N conc.	0.632***	−0.614***	0.070	0.635***	0.618***	−0.286
P conc.	0.392*	0.697***	−0.326	0.484**	0.812***	−0.558**
N:P ratio	0.050	−0.442*	0.655***	−0.027	−0.489**	0.547**

Data are correlation coefficients. n = 8 for foliage; n = 32 for L and F/H layers and fine roots. * *P*<0.05; ** *P*<0.01; *** *P*<0.001.

### Nitrogen and P contents and N:P ratio

Soil total N:P ratios and extractable N:P ratios varied across the eight study sites, both were lower at the PF site (5.4 and 1.8, respectively) and REB1 site (5.5 and 1.8, respectively) than at other sites (7.2 to 11.0 and 3.0 to 6.1, respectively; Table 4). The PF site, with a long history of human disturbance, was distinct from other sites, with higher bulk density and lower organic C, total N and extractable N pools in the 0–15 cm mineral soil (Table 4). Soil total P and extractable P pools at the PF site were also lower than at the old-growth forest sites (REB1 and 2, MEB and MTEB), but comparable to those at other mature forest sites (PBM1, 2 and 3; Table 4).

**Table 4:** Selected characteristics of the 0–15 cm mineral soil.

Site	Bulk density (g/cm^3^)	Soil organic C pool (Mg/ha)	Soil total N pool (kg/ha)	Soil total P pool (kg/ha)	Soil total N:P ratio	Soil extractable N pool (kg/ha)	Soil extractable P pool (kg/ha)	Soil extractable N:P ratio
PF	1.35(0.02)	17(2)	1148(150)	213(13)	5.4(0.6)	36(3)	22(3)	1.8(0.3)
PBM1	0.92(0.03)	35(1)	1759(77)	200(11)	8.8(0.3)	81(5)	17(2)	4.8(0.6)
PBM2	1.02(0.07)	31(5)	1774(271)	243(29)	7.2(0.3)	96(6)	17(3)	6.1(1.2)
PBM3	0.98(0.04)	33(2)	2023(115)	185(12)	11.0(0.7)	91(8)	19(1)	4.9(0.2)
REB1	1.09(0.04)	28(2)	2156(201)	394(36)	5.5(0.2)	117(10)	65(6)	1.8(0.1)
REB2	0.89(0.04)	43(4)	2789(266)	330(15)	8.4(0.7)	113(14)	39(4)	3.0(0.5)
MEB	0.84(0.04)	40(4)	2682(268)	321(19)	8.4(0.7)	128(13)	33(3)	3.9(0.4)
MTEB	0.88(0.03)	44(3)	2533(178)	280(16)	9.0(0.4)	123(11)	25(4)	5.1(0.8)

All data are means (±1 SE), n = 4. The corresponding full names of eight study sites are listed in Table S1.

**Figure 5:**
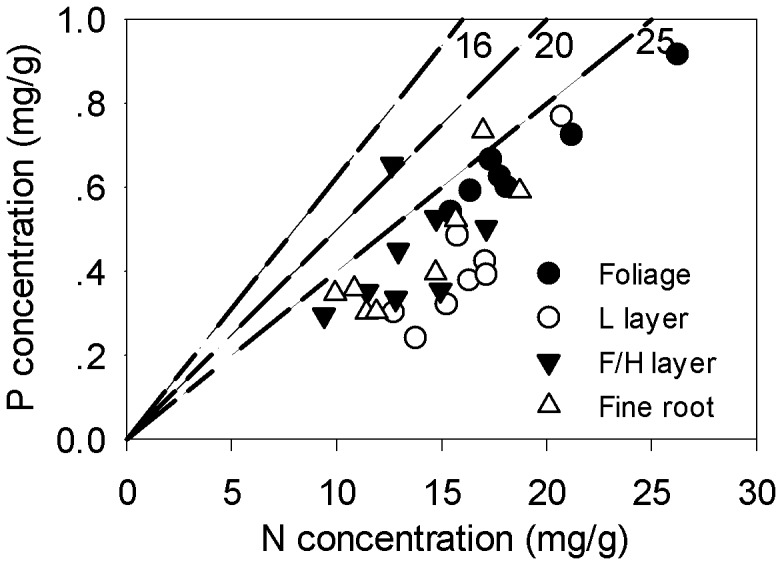
Mean N and P concentrations of plant materials of eight study forests. Dashed lines depict N:P ratios of 16, 20 and 25 on a mass basis. Ratios of 16 and 20 are P limitation thresholds of plant growth proposed by Koerselman and Meuleman (1996) [34] and Güsewell (2004) [31], respectively; ratio of 25 is the critcial N:P ratio that indicates P limitation on litter decomposition proposed by Güsewell and Verhoeven (2006) [61].

Nitrogen and P concentrations varied widely in all plant materials, with average N concentrations of 21.0 mg/g, 16.1 mg/g, 13.2 mg/g and 13.8 mg/g, respectively in the foliage, L and F/H layers and fine roots, while corresponding values for P in these materials were 0.77 mg/g, 0.41 mg/g, 0.43 mg/g and 0.44 mg/g, respectively (Table 5). Site averages of foliar N concentration were all above 15 mg/g (Table S3), and species averages were all higher than 15 mg/g as well, except for the *Pinus massoniana* (14.6 mg/g; from PF site) and *Rhododendron henryi* (11.2 mg/g; from MTEB site; Table S4). Although N:P ratio also varied widely in all plant materials, the values (15.6–72.0) were generally high (Table 5), with 30 of the 32 site averages higher than 25 and other two values around 16 and 20 (Figure 5 and Table S3).

**Table 5:** Statistical characteristics of nutrient measures of selected plant materials.

	N concentration (mg/g)		P concentration (mg/g)		N:P ratio	
Material	Mean	Range	Mean	Range	Mean	Range
Foliage	21.0(0.8)	10.7–43.9	0.77(0.04)	0.37–1.97	28.3(0.6)	15.8–41.4
L layer	16.1(0.5)	11.8–25.0	0.41(0.03)	0.21–0.89	42.3(2.0)	23.2–72.0
F/H layer	13.2(0.5)	8.2–19.5	0.43(0.02)	0.27–0.70	32.0(1.5)	15.6–58.7
Fine roots	13.8(0.7)	8.9–21.8	0.44(0.03)	0.24–0.88	32.7(1.2)	21.5–46.1

Data in the brackets are SE; n = 96 for foliage, = 32 for L and F/H layers and fine roots.

## Discussion

### Overall Relationship Patterns

Our results revealed consistent relationship patterns of community biomass, litterfall C, N and P productions, forest floor turnover rate and microbial properties with measures of N and P as well as N:P ratios (Figures 2, 3 and 4 and Table 1) that generally suggested that P availability played a more significant role than N availability in determining ecosystem primary productivity and nutrient cycling at the study sites. Considering that ecosystem productivity increases with increasing supply of a limiting nutrient, while showing no or even a negative response to the increased supply of a non-limiting nutrient [30], [59], [60], our results suggest that P availability was one of the limiting factors of plant growth and nutrient cycling at the study sites.

The idea is supported by the high N:P ratios of foliage, forest floors and fine roots at these sites. Site averages of the N:P ratios of these plant materials (30 of the 32 values higher than 25; Figure 5 and Table S3) were mostly higher than the proposed breakpoints of P limitation on plant growth by both Koerselman and Meuleman (1996) [34] (N:P ratio = 16) and Güsewell (2004 ) [31] (N:P ratio = 20), and also higher than the critical ratio that indicate P limitation on the decomposition of graminoid leaf litter (N:P ratio = 25) [61]. Moreover, average N:P ratios of foliage (28.3) and forest floors (L layer 42.3; F/H layer 32.0) in this study were much higher than the global averages of both temperature (broadleaf foliage 12.8, coniferous foliage 9.8; broadleaf litter 13.2, coniferous litter 11.8) and tropical regions (foliage 19.6, litter 28.4) [6]. These results generally suggest strong P limitation on primary productivity and litter decomposition at the study sites.

Our results are consistent with previous studies in which experimental N additions (50, 100 and 150 kg N ha^−1^ yr^−1^) generally did not increase (and in some cases actually decreased) the understory vegetation biomass, litter decomposition rate and soil respiration rate at three (PF, PBM2 and MEB) of the eight study sites [25], [26], [62], [63]. Experimental P addition (150 kg P ha^−1^ yr^−1^) significantly increased soil microbial biomass C concentration, soil respiration and litterfall production at the old-growth MEB site, and also significantly increased the litterfall production at the other two sites (PF, PBM2) [27].

### Different Relationship Patterns for Different Study Sites and Ecosystem Compartments

Although the relationship patterns suggested significant P limitation at the study sites in general, ecosystem productivity or processes may differ in the extent of P limitation or may be co-limited by N at different sites. Fertilization studies showed that at the old-growth MEB site, understory vegetation biomass, litter decomposition rate and soil respiration all responded negatively to experimental N additions [62], [26], and soil microbial biomass and respiration and litterfall production showed positive responses to experimental P addition [27]. In contrast, at the PF site, understory vegetation biomass, litter decomposition and soil respiration at the PF site did not respond to the N additions [25], [62], soil microbial biomass and respiration also did not respond to the P addition [27]. The responses of the PBM2 site to fertilization additions generally fell between the MEB site and PBM2 site [25]–[27], [62].

The different nutrient limitation patterns of different sites were also reflected by our plots in this study. For example, plot of community biomass against soil extractable N:P ratio showed a distinct pattern at the PF site as compared to the other sites (Figure 2F). For sites with similar community biomass, soil extractable N:P ratio of the PF site was much lower than that of other sites. The distinct pattern was largely attributed to the markedly lower soil extractable N pool of the PF site than that of other sites (Table 4 and Figure 2D). Similarly, the plot of total N:P ratio against total P concentration in the soil also showed a distinct pattern of the PF site from other sites (Figure S2). For soils with similar total P concentration, total N:P ratio of the PF site was much lower than that of other sites due to its lower soil total N concentration compared with other sites. These distinct patterns of the PF site were due to its low soil N availability that probably because of the continuous removal of a large amount of nutrients, particular of N, by the prescribed over 40 years (1950s–1990s) of understory and litter harvest at this site [45].

Several recent studies have found that different ecosystem compartments or processes may differ in response to the addition of the same nutrient [27], [64], [65]. Nutrient limitation of one ecosystem compartment or process cannot be simply predicted from nutrient limitation of another ecosystem compartment or process [60], [64], [65]. In this study, community biomass was more strongly related to soil extractable P pool than litterfall C production (Figures 2E and 3B), suggesting a greater constraint of P availability on community biomass than on litterfall C production. This was probably because of the indirect impact of P availability on litterfall C production via its impact on community biomass, as supported by significant relationship of litterfall C production with community biomass (R^2^ = 0.88, *P* = 0.014, n = 7; data not shown).

Similarly, the relationships of litterfall C, N and P productions with soil P pools were also different. Stronger relationships of litterfall P and N productions with soil extractable P pool (R^2^ = 0.76 and 0.47, respectively; Figures 3D and F) than litterfall C production (R^2^ = 0.18; Figure 3B) suggest a stronger impact of soil P availability on litter chemistry than on litter quantity. Significant relationships between N and P concentrations and N:P ratio of forest floors and soil P concentrations (Table 2) also suggested the significant impacts of P availability on litter chemistry. These results are consistent with several other studies which revealed that forests on low-fertility soils tended to produce similar quantities of litters as forests on high-fertility soils nearby, but of lower quality [4], [5], [66]–[68].

Climate conditions (e.g. evapotranspiration) are likely to be the major factor affecting litter decomposition at a global scale, while at a local scale litter chemistry is always the major factor affecting litter decomposition [14], [69], [70]. In this study, forest floor turnover rate, microbial biomass C concentration, microbial respiration and β-glucosidase activity were all significantly related to the nutrient measures of the forest floors, with stronger relationships with the P concentration than with the N concentration and negative relationships with the N:P ratio in either L layer or F/H layer or both layers. As for the previous patterns these results suggest P availability rather than N availability plays an important role in controlling litter decomposition rates at all levels from ecosystem, to microbes and enzymes.

### Constraint of Ecosystem Development by Physical Environment

The species composition of natural plant communities develops over a long period of succession. During succession, the community interacts with the physical environment (e.g. light, temperature and soil fertility) and finally reaches a relative steady status [71]–[73]. Physical environments may determine how far a community goes [71], [73], [74]. At the global or climate scale, primary productivity of forest ecosystems is largely determined by the climate conditions (e.g. precipitation) [75], [76]. While at a local scale, if light, temperature and rainfall are relatively consistent across different terrestrial communities, soil fertility may be the major physical condition determining the mature stage of succession [1], [2], [14]. In this study, the close relationships of community biomass with soil nutrient pools (both total and extractable) did support this hypothesis. Soil total and extractable P pool explained 39% and 59% of the community biomass variation, respectively (Figures 2B and E).

Despite strong theoretical predictions suggesting the existence of relationship between community biomass and soil fertility [14], [71], [74], the relationships of community biomass and productivity with soil nutrients have been frequently poor in natural ecosystems [77], [78], particular in forest ecosystems with high plant diversity as our study forests [14], [79]. We proposed that the strong relationship of community biomass and litterfall N and P productions with soil P pools were probably because of markedly P limitation and N enrichment at the study sites compared with many other areas.

In addition to the high foliar and litter N:P ratios, mean N concentrations of the foliage (21.0 mg/g) and L layer (16.1 mg/g) were both ca. 2 times higher than the global averages for evergreen trees and shrubs (foliage 13.7 mg/g; litter 7.3 mg/g) [80], while mean P concentrations (foliage 0.77 mg/g; L layer 0.41 mg/g) were lower than reported global averages (foliage 1.02 mg/g and litter 0.50 mg/g) [80]. Moreover, foliar N concentrations of 11 of the 13 species in this study (range 17.1–42.1 mg/g, the other two values were 11.2 mg/g and 14.6 mg/g; Table S4) were in the upper range of species-specific foliar N concentrations of the tropical rain forests (mostly in the range of 10–30 mg/g) [35], and L layer N concentrations at six of the eight study sites (range of 15.2–20.7 mg/g, the other two values were 12.7 mg/g and 13.7 mg/g; Table S3) were in the upper ranges of site averages of litter N concentration of the tropical rain forests (mostly in the range of 10–19 mg/g) [67]. These results all suggest relatively high N availability and low P availability at our study sites compared with many other areas, which may underlie the strong relationships found between community biomass and litterfall N and P productions and soil P pools in this study.

### How do these Consistent Patterns Occur?

As proposed above, strong limitation of P and enrichment of N might be the main causes of the consistent patterns observed in this study. The causes of P limitation in terrestrial ecosystems can be complicated. Here we only address three of six pathways proposed by Vitousek et al. (2010). First is the pathway of depletion that is likely to occur during millions of years of soil development [18], [81], [82]. Soils at the study sites developed during the Holocene (<15 ky) [39]. The soil ages are comparable to the Laupahoehoe site (20 kyr) in Hawaii that was found to be co-limited by N and P in fertilization studies [82]. The Laupahoehoe site is at comparable latitude (20°N), with similar mean annual temperature (16?), precipitation (2500 mm) and elevation (1170 m) [83] as our study sites (latitude 23°N; mean annual temperature 21?; precipitation 1900 mm; and elevation 50–600 m). Therefore, soil age is not likely to be the major cause of P limitation at the study sites. The second proposed pathway is low-P parent material that can cause P limitation developed quickly and persist over a long timescale [18]. Phosphorus concentrations in the C layer of the study area are approximately 0.40 mg/g [84], which are lower than the average in continental crust (0.70 mg/g, range of 0.04–3.00 mg/g) [18]. Therefore, low P concentration in the parent rock appears to contribute to the P limitation at the study sites but is not likely to be a major cause either.

The third possible pathway is anthropogenic P limitation by enhanced supply of other resources (especially N) [16]–[18]. This pathway is probably the most important cause of P limitation at the study sites, since N availability at the study sites was high compared with many other areas in the world as discussed above. The high N availability may be mainly attributed to two pathways: accumulation of N through biological fixation during long-term (>80 years old) forest development [28], [72], and high N deposition at the study sites [42], [85]. Given an annual atmospheric N input of 40 kg N ha^−1^ yr^−1^ [42], [85], atmospheric N input have accounted for 16% to 47% (mean 27%) of the annual total N inputs to the soils (atmospheric N input plus litterfall N production) at the study sites during the last two decades. A recent study reported the gradual increase of vegetation N:P ratios from 21 to 28 at the MEB site during the last three decades and a significant increase of foliar N:P ratio after 100 and 150 kg ha^−1^ yr^−1^ N added [42]. Together these lines of evidence suggested the possibility that the sites were driven to P limitation by excessive N inputs at the study sites.

### Conclusions

Our study has revealed consistently stronger relationships between measures of P availability and community biomass, litterfall C, N and P productions, forest floor turnover rates and litter chemistry than we found for measures of N availability, indicating a significant role of P in determining ecosystem primary productivity and processes at the study sites. Vegetation N:P ratios indicated strong P limitation at all sites in this study. The results also showed that different ecosystem compartments or processes may differ in the extent of the nutrient limitation at different sites that depend on the soil nutrient status and land use history of these sites. In general, these results suggested constraint of ecosystem development by soil P availability at the study sites. We proposed that markedly N enrichment was probably a significant driver of the strong P limitation at these study sites. Low P parent material may also partly contribute to the P limitation. Further study is warranted on the mitigation of P limitation of ecosystem productivity and processes in the tropical and subtropical China.

## Supporting Information

Figure S1 Correlations between N and P concentrations and N:P ratio of the foliage samples.
(TIF)

Figure S2 Correlations between total N and total P concentrations and total N:P ratio of the 0–15 cm mineral soil.
(TIF)

Table S1 Characteristics of eight study forest sites at Dinghushan Biosphere Reserve, China.
(DOC)

Table S2 Litterfall C production, forest floors biomass C, and forest floor turnover rate of eight study forests.
(DOC)

Table S3 Site averages of N and P concentrations and N:P ratio of foliage, L and F/H layers and fine roots of eight study forests.
(DOC)

Table S4 Species averages of foliar N and P concentrations and mass-based N:P ratio of 13 tree species selected from eight study forests.
(DOC)
