# Supplementary material for: Nutrient Limitation on Ecosystem Productivity and Processes of Mature and Old-Growth Subtropical Forests in China
Source: PLoS One. 2012 Dec 20;7(12):e52071. doi: 10.1371/journal.pone.0052071 (PMC3527367; doi:10.1371/journal.pone.0052071)
Supplement: Table S1 — Characteristics of eight study forest sites at Dinghushan Biosphere Reserve, China. (DOC) [file pone.0052071.s003.doc]

**Table S1.** Characteristics of the eight study forest sites at Dinghushan Biosphere Reserve, China.

| Site | Site full name | Succession stage | Age (year) | Aspect | Slope | Altitude | Major tree species | Community biomass ※ (Mg/ha) | Litterfall production§ (Mg^-1^·ha^-1^·yr) |
| --- | --- | --- | --- | --- | --- | --- | --- | --- | --- |
| PF | Pine forest | Pioneer community | 80 | SE | 25° | 50-150m | *Pinus massoniana*, *Schima superba* and *Euodia lepta* | 122.5 | 3.56 |
| PBM1 | Pine and broadleaved mixed forest 1 | Transition community | 80 | SE | 30° | 50-200m | *Pinus massoniana*, *Schima superba* and *Castanea henryi* | 265.6 | 8.61 |
| PBM2 | Pine and broadleaved mixed forest 2 | Transition community | 80 | NE | 40° | 150-200m | *Pinus massoniana*, *Schima superba* and *Castanea henryi* | 164.1 | 7.01 |
| PBM3 | Pine and broadleaved mixed forest 3 | Transition community | 80 | NE | 25° | 300-350m | *Pinus massoniana*, *Schima superb* and *Castanea henryi* | 276.8 |  |
| REB1 | Ravine evergreen broadleaved forest 1 | Topographical climax | 300 | E | 20-25° | 200-250m | *Aporusa yunnanensis*, *Cryptocarya concinna* and *Ormosia fordiana* | 472.9 | 10.16 |
| REB2 | Ravine evergreen broadleaved forest 2 | Topographical climax | 300 | W | 30-40° | 100-150m | *Gironniera subaequalis*, *Sterculia lanceolata* and *Caryota ochlandra* | 391.2 | 10.61 |
| MEB | Monsoon evergreen broadleaved forest | Regional climax | >400 | NE | 30° | 250-300m | *Schima superba*, *Castanea henryi* and *Cryptocarya concinna* | 290.4 | 8.49 |
| MTEB | Mountainous evergreen broadleaved forest | Topographical climax | >100 | NE | 30° | 550-600m | *Englhardtia roxburghiana*, *Rhododendron henryi* and *Machilus breviflora* | 165.1 | 4.89 |

※Community biomass includes dry weight of whole plants of all trees and shrubs with diameter at breast height (DBH) ≥ 1cm at permanent plots with areas ranging from 1000 to 10000 m^2^ [1]. They were calculated from the investigated DBH during 1996-2004 using the regression equations for biomass estimation of Wen et al. (1997) [1,2]. The regression equations were constructed by building the relationship between dry weight and DBH of 44 shrubs or trees whole-harvested from a destructive plot nearby the MEB site at four levels of DBH (DBH ≤ 5 cm, n=10; 5 < DBH ≤ 10, n=12; 10 < DBH ≤ 20; n=13; DBH > 20 cm; n= 9) [2]. For sites with several times of investigation, the latest data was used in this study (MEB, MTEB, PBM1, PBM2 and PBM at 2004; PBM3 at 2002; REB2 at 2001; REB1 at 1996) [1].

§ Averages of litterfall production during 1981-2001 for all sites [3], except for the PBM2 site which was from one year measurement (March 2003 to April 2004) [4]. No data for the PBM3 site. Fifteen aboveground litterfall traps of 1 m^2^ were randomly spread at a 1 to 2 ha plot of each community for litter collection; litterfall was collected once a month [3].

**References**

1. Liu S, Luo Y, Huang YH, Zhou GY (2007) Studies on the community biomass and its allocations of five forest types in Dinghushan Nature Reserve. Ecol Sci 26: 387-393.
2. Wen DZ, Wei P, Kong GH, Zhang QM, Huang ZL (1997) Biomass study of the community of *Castanopsis chinensis* + *Cryptocarya concinna* + *Schima superba* in a southern China reserve. Acta Ecol Sinica 17: 497-504.
3. Zhou GY, Guan LL, Wei XH, Zhang DQ, Zhang QM, et al. (2007) Litterfall production along successional and altitudinal gradients of subtropical monsoon evergreen broadleaved forests in Guangdong, China. Plant Ecol 188: 77-89.
4. Yan JH, Zhang DQ, Zhou GY, Liu JX (2009) Soil respiration associated with forest succession in subtropical forests in Dinghushan Biosphere Reserve. Soil Biol Biochem 41: 991-999.
