# Supplementary material for: Nutrient Limitation on Ecosystem Productivity and Processes of Mature and Old-Growth Subtropical Forests in China
Source: PLoS One. 2012 Dec 20;7(12):e52071. doi: 10.1371/journal.pone.0052071 (PMC3527367; doi:10.1371/journal.pone.0052071)
Supplement: Table S2 — Litterfall C production, forest floors biomass C, and forest floor turnover rate of eight study forests. (DOC) [file pone.0052071.s004.doc]

**Table S2. Litterfall C production, forest floors biomass C, and forest floor turnover rate of eight study forests.**

| Site | Litterfall C production  (Mg^-1^ ha^-1^ yr) | L layer biomass C (Mg/ha) | F/H layer biomass C (Mg/ha) | Forest floor biomass C (Mg/ha) | Forest floor turnover rate (yr^-1^) |
| --- | --- | --- | --- | --- | --- |
| PF | 1.8 | 2.1(0.2) | 15.3(1.7) | 17.4(1.7) | 0.10 |
| PBM1 | 4.3 | 1.7(0.0) | 5.1(0.7) | 6.8(0.6) | 0.63 |
| PBM2 | 3.4 | 1.8(0.3) | 4.4(0.7) | 6.2(0.8) | 0.55 |
| PBM3 | N/A | 1.1(0.2) | 3.3(0.2) | 4.3(0.2) | N/A |
| REB1 | 4.3 | 0.6(0.1) | 2.7(0.8) | 3.3(0.9) | 1.30 |
| REB2 | 4.5 | 0.6(0.0) | 2.0(0.2) | 2.6(0.2) | 1.75 |
| SSEB | 4.0 | 1.3(0.1) | 2.6(0.3) | 3.9(0.3) | 1.02 |
| MTEB | 2.4 | 0.8(0.1) | 2.7(0.2) | 3.5(0.3) | 0.67 |

Data in bracket are SE, n = 4. N/A indicates no data. Litterfall C production = litterfall productivity × L layer C concentration. Forest floor turnover rate = litterfall C production / forest floor biomass C. See Table S1 for full names of the sites.
