# Supplementary material for: Nutrient Limitation on Ecosystem Productivity and Processes of Mature and Old-Growth Subtropical Forests in China
Source: PLoS One. 2012 Dec 20;7(12):e52071. doi: 10.1371/journal.pone.0052071 (PMC3527367; doi:10.1371/journal.pone.0052071)
Supplement: Table S3 — Site averages of N and P concentrations and N:P ratio of foliage, L and F/H layers and fine roots of eight study forests. (DOC) [file pone.0052071.s005.doc]

**Table S3**. Site averages of N and P concentrations and N:P ratio of foliage, L and F/H layers and fine roots of eight study forests.

| Plant material | Site | n | N concentration (mg/g) | P concentration (mg/g) | N:P ratio |
| --- | --- | --- | --- | --- | --- |
| Foliage | PF | 12 | 21.2(2.5) | 0.73(0.06) | 29.8(2.7) |
|  | PBM1 | 12 | 17.3(0.7) | 0.67(0.04) | 27.0(2.0) |
|  | PBM2 | 12 | 17.7(0.6) | 0.63(0.04) | 29.4(1.8) |
|  | PBM3 | 12 | 16.4(0.7) | 0.59(0.02) | 27.9(1.4) |
|  | REB1 | 12 | 26.2(2.5) | 0.92(0.06) | 28.4(1.4) |
|  | REB2 | 12 | 35.7(1.4) | 1.51(0.11) | 25.8(2.9) |
|  | SSEB | 12 | 18.1(0.7) | 0.60(0.02) | 30.1(0.7) |
|  | MTEB | 12 | 15.4(0.9) | 0.54(0.03) | 28.5(0.6) |
| L layer | PF | 4 | 12.7(0.3) | 0.30(0.02) | 42.2(2.0) |
|  | PBM1 | 4 | 13.7(0.9) | 0.24(0.02) | 57.0(1.1) |
|  | PBM2 | 4 | 16.3(0.3) | 0.38(0.02) | 43.1(1.4) |
|  | PBM3 | 4 | 15.2(0.6) | 0.32(0.01) | 47.3(1.3) |
|  | REB1 | 4 | 20.7(1.6) | 0.77(0.05) | 27.1(1.9) |
|  | REB2 | 4 | 15.7(1.2) | 0.49(0.05) | 33.9(5.4) |
|  | SSEB | 4 | 17.0(2.1) | 0.43(0.07) | 43.7(9.9) |
|  | MTEB | 4 | 17.1(0.9) | 0.39(0.02) | 43.8(2.7) |
| F/H layer | PF | 4 | 14.9(0.5) | 0.36(0.02) | 42.2(1.7) |
|  | PBM1 | 4 | 11.4(0.9) | 0.35(0.01) | 32.4(1.8) |
|  | PBM2 | 4 | 17.1(1.1) | 0.50(0.03) | 34.1(1.9) |
|  | PBM3 | 4 | 9.4(0.4) | 0.30(0.02) | 32.2(3.0) |
|  | REB1 | 4 | 12.6(1.2) | 0.65(0.03) | 19.3(1.4) |
|  | REB2 | 4 | 14.7(0.7) | 0.53(0.04) | 28.1(0.9) |
|  | SSEB | 4 | 12.9(1.0) | 0.45(0.02) | 28.7(1.9) |
|  | MTEB | 4 | 12.8(1.3) | 0.34(0.02) | 39.4(6.7) |
| Fine roots | PF | 4 | 10.8(0.4) | 0.36(0.03) | 30.7(1.6) |
|  | PBM1 | 4 | 11.4(1.3) | 0.30(0.03) | 38.1(1.9) |
|  | PBM2 | 4 | 9.9(0.3) | 0.35(0.03) | 29.0(2.0) |
|  | PBM3 | 4 | 11.9(1.1) | 0.30(0.03) | 39.8(3.2) |
|  | REB1 | 4 | 17.0(1.1) | 0.73(0.05) | 23.2(1.0) |
|  | REB2 | 4 | 18.7(1.6) | 0.59(0.08) | 33.1(4.5) |
|  | SSEB | 4 | 15.7(1.9) | 0.52(0.05) | 30.2(3.1) |
|  | MTEB | 4 | 14.7(1.1) | 0.40(0.04) | 37.6(1.7) |

Data are means with SE in the bracket. See Table S1 for the full name of the sites.
