# Supplementary material for: Nutrient Limitation on Ecosystem Productivity and Processes of Mature and Old-Growth Subtropical Forests in China
Source: PLoS One. 2012 Dec 20;7(12):e52071. doi: 10.1371/journal.pone.0052071 (PMC3527367; doi:10.1371/journal.pone.0052071)
Supplement: Table S4 — Species averages of foliar N and P concentrations and mass-based N:P ratio of 13 tree species selected from eight study forests. (DOC) [file pone.0052071.s006.doc]

**Table S4**. Species averages of foliar N and P concentrations and mass-based N:P ratio of 13 tree species selected from eight study forests.

| Species | n | N concentration (mg/g) | P concentration (mg/g) | N:P ratio |
| --- | --- | --- | --- | --- |
| *Gironniera subaequalis* | 4 | 42.1(0.8) | 1.08(0.03) | 39.0(1.4) |
| *Ormosia fordiana* | 4 | 37.5(1.5) | 1.18(0.05) | 32.2(2.8) |
| *Caryota ochlandra* | 4 | 33.4(1.2) | 1.77(0.06) | 18.9(1.0) |
| *Euodia lepta* | 4 | 32.7(0.8) | 0.95(0.05) | 34.8(1.6) |
| *Sterculia lanceolata* | 4 | 31.6(0.3) | 1.68(0.17) | 19.4(2.0) |
| *Cryptocarya concinna* | 8 | 21.0(0.3) | 0.69(0.01) | 30.7(0.7) |
| *Aporusa yunnanensis* | 4 | 20.4(0.7) | 0.87(0.04) | 23.4(0.3) |
| *Castanea henryi* | 16 | 18.1(0.4) | 0.61(0.02) | 29.6(0.6) |
| *Engelhardia fenzelii* | 4 | 18.0(0.3) | 0.63(0.03) | 28.8(0.9) |
| *Schima superba* | 20 | 17.5(0.3) | 0.53(0.01) | 33.3(0.8) |
| *Machilus breviflora* | 4 | 17.1(0.4) | 0.60(0.02) | 28.8(1.7) |
| *Pinus massoniana* | 16 | 14.6(0.4) | 0.73(0.03) | 20.2(0.7) |
| *Rhododendron henryi* | 4 | 11.2(0.3) | 0.40(0.02) | 27.9(0.4) |

*Note*: Data are mean with SE in the bracket.
